# Clinical efficacy of Danzhi Xiaoyao Powder in the treatment of post-stroke depression: A protocol for randomized, double-blind clinical study

**DOI:** 10.1097/MD.0000000000027318

**Published:** 2021-10-22

**Authors:** ChunQin Ding, MingYang Xu, Li Gao, XiaoRong Wang, Wu Xu, MinWang Guo, Jun Yao

**Affiliations:** Taixing People's Hospital, Department of Neurology, No.1 Changzheng Road, Taixing, Jiangsu Province, P.R. China.

**Keywords:** danzhi xiaoyao powder, post-stroke depression, protocol, randomized controlled trial

## Abstract

**Background:**

Depression is a common complication after stroke and is closely related to the poor prognosis of stroke. Antidepressants are the priority drug in the treatment of post-stroke depression (PSD), but there are dependence and adverse reactions. Danzhi Xiaoyao Powder has a good effect on depression without obvious adverse reactions. At present, there is a lack of rigorous randomized controlled trials to evaluate the clinical efficacy of Danzhi Xiaoyao Powder in the treatment of PSD.

**Methods:**

This is a prospective, randomized, double-blind, parallel controlled trial to explore the efficacy and safety of Danzhi Xiaoyao Powder in the treatment of PSD. The participants were randomly divided into treatment group and control group. The treatment group used Danzhi Xiaoyao Powder combined with escitalopram oxalate, and the control group used Danzhi Xiaoyao Powder simulant combined with citalopram oxalate. The two groups were both treated for 8 weeks and followed up for 3 months. Observational index includes: Total response rate, Hamilton depression scale, Barthel index, national institutes of health stroke scale, the modified Edinburgh-Scandinavian stroke scale, Incidence of adverse reactions. Finally, SPASS 22.0 software was used for statistical analysis of the data.

**Discussion:**

This study will evaluate the clinical efficacy of Danzhi Xiaoyao Powder in the treatment of PSD. The results of this study will provide reliable evidence for the clinical use of Xiaoyao Powder in the treatment of PSD.

**Trial registration:**

Open Science Framework Registration number: DOI 10.17605/OSF.IO/5V926

## 1 Introduction

Stroke is one of the major causes of death and disability worldwide,^[1]^ and depression is a common mental disorder after stroke.^[2]^ Post-stroke depression (PSD) is often accompanied by cognitive impairment, which adversely affects the recovery of patients and has become the most serious factor leading to poor quality of life of patients.^[3]^ The incidence of PSD is 29% to 31%,^[4]^ which usually occurs within 1 year after stroke.^[5]^ PSD is closely related to poor prognosis of stroke, which can not only lead to prolonged hospitalization, neurological recovery disorder, loss of independent living ability, but also cause increase of mortality.^[6–8]^ Studies have shown that the mortality of patients with PSD is significantly higher than that of stroke alone, the mortality of the former is 1.28 to 1.75 times the latter, and the severity of depression is highly correlated with the mortality.^[9]^

Currently, antidepressants are the priority drug for PSD. Although clinical studies have confirmed that antidepressants are effective for PSD,^[10,11]^ these drugs require long-term use and are prone to dependence and many adverse reactions.^[12]^ These negative factors may cause PSD patients or clinicians to explore alternative treatment options. Therefore, better treatment strategies for patients with PSD are crucial.

Traditional Chinese medicine has advantages of multi-target and multi-pathway, which plays an important role in complementary and alternative therapies, and has accumulated rich experience in the practice of treating PSD.^[13,14]^ Danzhi Xiaoyao Powder is a representative prescription for the treatment of mental diseases in traditional Chinese medicine. It is composed of Bai Zhu, Chai Hu, Dang Gui, Fu Ling, Gan Cao, Dan Pi, Zhi Zi, and Bai Shao, which has the function of dispersing stagnated liver qi for relieving qi stagnation and regulating qi-flowing for promoting blood circulation.^[15]^ It was found that Danzhi Xiaoyao Pill could regulate the metabolism of phenylalanine, arachidonic acid, porphyrin, D-arginine and D-ornithine, adjust steroid biosynthesis and unsaturated fatty acid biosynthesis to increase the excitability of the body and play the role of anti-depression.^[16]^ Some clinical studies have also confirmed that Danzhi Xiaoyao Powder can reduce HAMD score, increase neurotransmitter power and 5-hydroxytryptophan level in patients with PSD, without increasing adverse reactions.^[17,18]^ At present, although some clinical studies have evaluated the efficacy of Xiaoyao Powder in treating PSD, the intervention and follow-up time of these studies are short, which cannot reliably evaluate the long-term efficacy and stability of Xiaoyao Powder in treating PSD. Also, there is a lack of rigorous placebo control to determine whether Xiaoyao Powder can reduce the adverse effects of antidepressants. Therefore, we will evaluate the efficacy and safety of Xiaoyao Powder in the treatment of PSD through this randomized, double-blind, parallel controlled study.

## 2 Materials and methods

### 2.1 Study design

**Figure 1:**
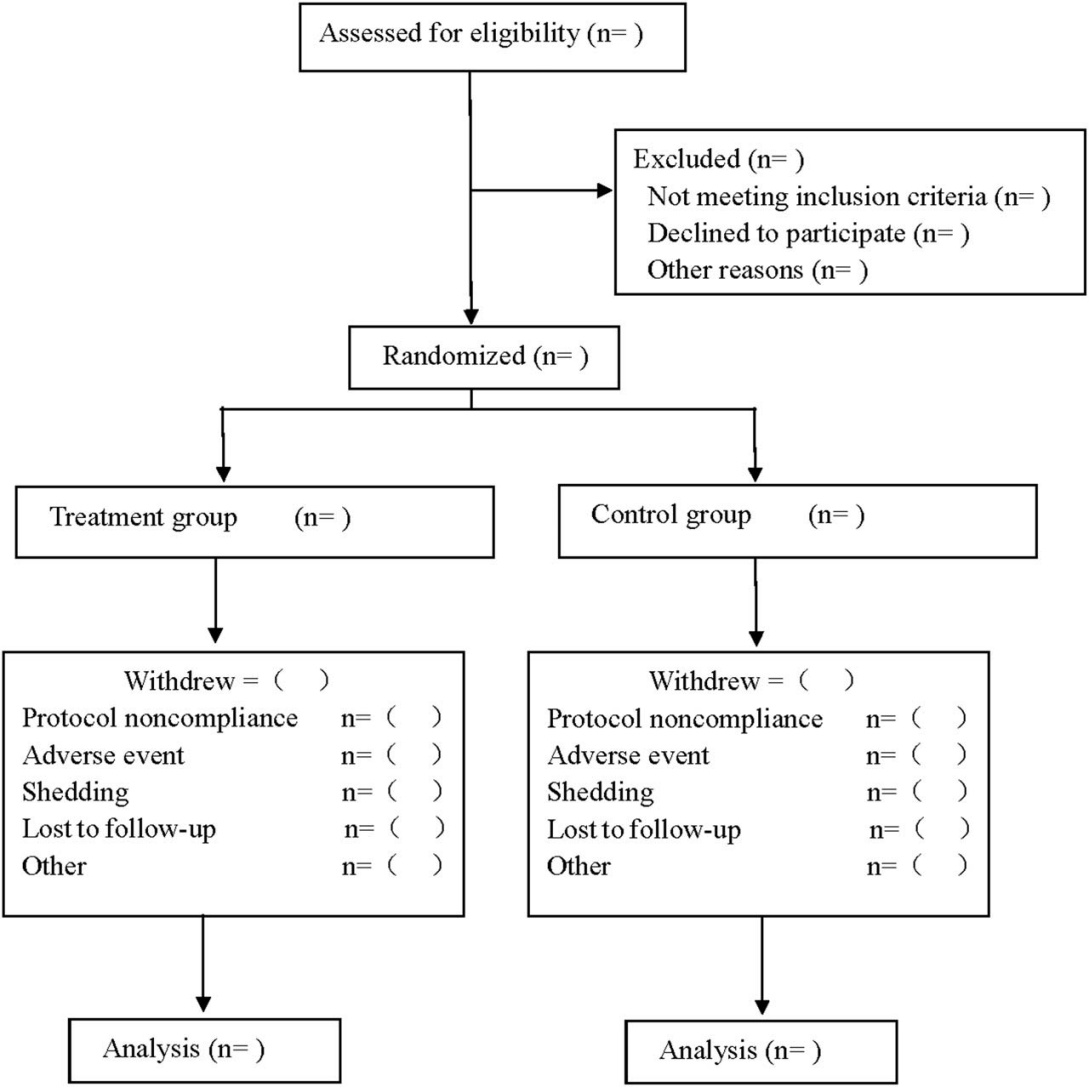
Flow diagram.

This trial was designed as a double-blind, randomized, controlled, and parallel-group study that focused on the therapeutic efficacy and safety of Danzhi Xiaoyao Powder combined with antidepressant in treating PSD. The participants were randomly divided into treatment group and control group. The treatment group used Danzhi Xiaoyao Powder combined with escitalopram oxalate, and the control group used Danzhi Xiaoyao Powder simulant combined with citalopram oxalate. The two groups were both treated for 8 weeks and followed up for 3 months. The research protocol followed the latest Consolidated Standards of Reporting Trials 2017 (Consolidated Standards of Reporting Trials 2017 is shown in Fig. 1), and Standard Protocol Items: Recommendations for Interventional Trials 2013 statement (Standard Protocol Items: Recommendations for Interventional Trials checklist see Supplementary Digital Content Table S1).

### 2.2 Ethics and registration

This Research protocol will be conducted in accordance with the Declaration of Helsinki and Ethical Guidelines for Clinical Research. This study has been approved by our Clinical Research Ethics Committee, and registered at open science framework (registration number: DOI 10.17605/OSF.IO/5V926). Before randomization, all patients were required to sign an informed consent form, so that they could choose whether to proceed with the experiment at any time.

### 2.3 Patients

#### 2.3.1 Diagnostic basis:

The diagnostic criteria for stroke refer to *Main Points of Diagnosis of Various Cerebrovascular Diseases*,^[19]^ for depression refer to the diagnostic criteria for PSD in *The Chinese Classification of Mental Disorders*,^[20]^ and for traditional Chinese medicine refer to *Diagnostic and Therapeutic Criteria of Traditional Chinese Medicine Diseases*.^[21]^

#### 2.3.2 Inclusion criteria:

(1) Patients whose age ≥18 years, and ≤75 years;
(2) Patients who meet the diagnostic criteria of stroke and depression, and Hamilton Depression Scale (HAMD) > 18 points;
(3) Patients who has not taken antidepressants (including Traditional Chinese medicine and Western medicine) in the last 2 weeks;
(4) Patients who agree to participate in this study and signed informed consent.

#### 2.3.3 Exclusion criteria:

(1) Patients with mental diseases other than depression;
(2) Patients who are addicted to alcohol, abuse and depend on psychoactive substances or drugs (including sleeping pills);
(3) Patients who cannot understand scale content due to consciousness or language barrier;
(4) Patients whose ALT, AST or Cr reaches 1.5 times of normal upper limit ^[22]^;
(5) Patients who are allergic to the investigational drug ingredients;
(6) Patients with severe mental diseases, unable to express themselves accurately or take medicine on time, or unable to complete the test.

#### 2.3.4 Shedding criteria and management:

(1) Patients who appear adverse reactions or serious adverse events (such as cardiovascular embolism, gastrointestinal reactions, severe liver and kidney dysfunction, etc.) or other complications. In that case, the test should be stopped and patients should be treated according to the judgment of the investigator;
(2) Patients who have poor compliance, change medical prescriptions half way or add other Chinese or Western drugs at will, especially using the combination of drugs that have a greater impact on the test drugs, which may affect the effectiveness and safety judgment;
(3) Patients who are unwilling or impossible to continue the clinical trial for any reason, and suspend the test by requesting the investigator to withdraw from the test;
(4) Patients who do not explicitly withdraw from the study, but no longer accepted drugs and detection.

For patients who dropped out of the trial or lost to follow-up, researchers should take active measures to complete the last test as far as possible, so as to analyze its efficacy and safety, and take corresponding treatment measures. All shedding cases should be recorded on the Case Report Form (CRF), and filled in the cause.

### 2.4 Sample size:

The sample size estimation was based on the results of the total response rate of the main efficacy indicators, referring to the results of pre-clinical trials, the total effective rate of Danzhi Xiaoyao Pill combined with escitalopram was 85%, and that of Danzhi Xiaoyao Powder simulant combined with escitalopram was 68%. PASS15.0 was used for sample size estimation, non-inferiority design was adopted, ɑ = 0.05, β = 0.1, test efficiency = 0.9, the number of cases in the treatment group: the number of cases in the control group = 1:1, critical value = -0.1. According to the software calculation, the total sample size of the two groups was 102 cases, considering the clinical shedding rate of about 10%, a total of 114 cases were finally included, with 57 cases in each group.

### 2.5 Randomization and blinding:

We will adopt a completely random method, using Excel 2007 software to generate random numbers and randomly divide them into two groups according to the ratio of 1:1. The random numbers will be put into 114 numbered sealed and opaque envelopes. The patients will randomly extract the envelopes and obtain the corresponding random numbers to complete the grouping. Throughout the study, patient statisticians and study participants were unknowable about the outcome of the randomized assignment.

### 2.6 Intervention measures:

Patients in both groups were given basic treatments such as hemostasis, anti-platelet aggregation, anti-infection, blood pressure and blood glucose regulation, and anti-brain edema according to *Guidelines for diagnosis and treatment of acute ischemic stroke in China (2018)*^[23]^ and *Guidelines for diagnosis and treatment of Cerebral hemorrhage in China* (2019).^[24]^

#### 2.6.1 Treatment group

(1) Danzhi Xiaoyao Powder (Bai Zhu 10 g, Chai Hu, 15 g, Dang Gui 10 g, Fu Ling 15 g, Gan Cao, 10 g, Dan Pi, 10 g, Zhi Zi, 10 g, Bai Shao, 15 g; made by Sichuan Neo-Green Pharmaceutical Technology Development Co., Ltd.), 1 bag at a time, 3 times a day; (2) Escitalopram oxalate (Janssen Pharmaceutical Ltd., Xi’an, China, J20100l65, 5 mg/tablet), twice a day, 5 mg for each oral administration.

#### 2.6.2 Control group

Danzhi Xiaoyao Powder simulator (made by Sichuan Neo-Green Pharmaceutical Technology Development Co., Ltd., its appearance and taste are the same as Xiaoyao Powder), 1 bag at a time, 3 times a day; (2) Escitalopram oxalate (Janssen Pharmaceutical Ltd., Xi’an, China, J20100l65, 5 mg/tablet), twice a day, 5 mg for each oral administration.

### 2.7 Outcomes:

#### 2.7.1 Primary outcome

(1) The total effective rate and curative effect were evaluated by the reduction rate of HAMD scores. The therapeutic criteria for depression were: recovery: reduction rate > 75%; significant effect: reduction rate > 50%; effective: reduction rate ≥25%; ineffective: reduction rate < 25%, total effective rate = (recovery number + significant effective number + effective number)/total number ∗100%; (2) HAMD scores.

#### 2.7.2 Secondary outcomes

(1) activities of daily living Barthel index; (2) national institutes of health stroke scale; (3) the modified Edinburgh-Scandinavian stroke scale; (4) Adverse reactions: including treatment-related discomfort experienced by patients during the study period.

### 2.8 Safety evaluation:

Blood routine, urine routine, liver function (ALT, AST) and renal function (urea nitrogen, creatinine) will be measured at baseline and after treatment to assess the safety of treatment. Patients were also asked to record any adverse reactions during the study and report them to the investigator at any time. Details of all adverse events will be recorded in the CRF, including time, extent, duration of occurrence, suspected reasons, effective measures and outcomes. After treatment, we will analyze the incidence of adverse reactions in both groups.

### 2.9 Data management and quality control:

Any modifications or changes to the protocol will be re-approved through a formal process by the hospital ethics committee. Independent clinical research assistants will periodically review research progress. Study data will be collected and recorded in CRF by trained investigators. To ensure reliability of data, personal information about potential and registered participants will be collected, shared and kept in a separate repository to protect confidentiality before, during and after the trial. Access to the database will be restricted to the researchers in the research team. Participants’ information will not be open or shared without their written permission.

### 2.10 Statistical analysis:

Efficacy evaluation will be determined by full analysis set and per-protocol set, and safety evaluation will be based on safety set. Statistical evaluation of full analysis set will follow intent-to-treat principles. Last observation Carried forward method was used to estimate missing values of major variables. The collected data were statistically analyzed by SPSS 22.0 software. Chi-square test was used for enumeration data; mean value ± standard deviation ($\bar{X}$ ± S) was used for measurement data, independent sample *t* test was used for normal distribution, and nonparametric test was used for skewed distribution. When *P* < .05, the difference was considered statistically significant.

## 3 Discussion

PSD refers to a series of psychological and physical syndromes represented by emotional depression, slow response, loss of interest and other symptoms after stroke.^[8]^ Stroke is an important social psychological factor leading to depression in patients, and neurological dysfunction and long-term disability caused by stroke lead to psychological stress response in patients, resulting in psychological imbalance.^[8]^ Depression goes against to the recovery of neurological function after stroke. Antidepressant treatment can not only improve the symptoms of depression, but also promote the physical recovery of stroke patients, whose significance is far beyond the depression treatment itself.^[14,25]^

After thousands of years of exploration, traditional Chinese medicine has had advantages in the treatment of psychological disorders.^[26]^ Danzhi Xiaoyao Powder is one of the most common traditional Chinese medicine prescriptions in the treatment of mental diseases. Clinical studies have found that Danzhi Xiaoyao Powder has the effect of psychotropic drugs, and has shown significant antidepressant effects in animal models.^[27]^ It could inhibit hyperactivity of hypothalamic-pituitary-adrenal and regulate monoamine and amino acid neurotransmitters in hippocampus. Chai Hu, Dang Gui and Bai Shao are the key drugs in many spiritual prescriptions, modern pharmacological studies have found that Chai Hu, Dang Gui, Bai Shao and other herbs in Xiaoyao Powder contain a variety of active ingredients, such as flavonoids, glycosides, phenols, polysaccharides, alkaloids, etc. These active ingredients all have obvious antidepressant activity. These active ingredients all have obvious antidepressant activity, which can effectively inhibit the reuptake of 5-hydroxytryptophan, norepinephrine, and dopamine in presynaptic membrane to achieve antidepressant effects.^[17]^ As there is no standard clinical study to evaluate the efficacy of Danzhi Xiaoyao Powder in the treatment of PSD, we intend to evaluate its efficacy and safety through a prospective randomized controlled study. HAMD score and its changes were used as the main outcome index to evaluate the efficacy of Danzhi Xiaoyao Powder on PSD, the effect of Danzhi Xiaoyao Powder on nerve recovery was evaluated by neurological function score, the effect of Danzhi Xiaoyao Powder on limb function recovery was evaluated by ADL score, and the safety of treatment was evaluated by adverse reactions and safety evaluation.

There are also some shortcomings in this study: as this study is a single-center study, it may lead to some single and regional samples; since we only observed the efficacy of acute gouty arthritis, the duration of medication was short, and patients were not allowed to take long-term medicine, and also there was no long-term follow-up visit, it was difficult to observe the occurrence of gout in the following two groups of patients.

## Author contributions

**Conceptualization:** ChunQin Ding and Li Gao.

**Data curation:** ChunQin Ding and MingYang Xu.

**Formal analysis:** Li Gao and XiaoRong Wang.

**Funding acquisition:** Jun Yao.

**Software:** XiaoRong Wang and Wu Xu.

**Supervision:** MingYang Xu and MinWang Guo.

**Writing – original draft:** ChunQin Ding and MingYang Xu.

**Writing – review & editing:** ChunQin Ding and Jun Yao.

## Supplementary Material

Supplemental Digital Content
